# Queerly departed: Queer viral socialities and Caribbean migrant desires

**DOI:** 10.1177/13634607231168992

**Published:** 2023-04-11

**Authors:** David AB Murray

**Affiliations:** York University, Toronto

**Keywords:** Caribbean, HIV/AIDS, migration, queer, sociality

## Abstract

In this paper I explore the transnational journeys of a group of queer HIV positive (HIV+) Caribbean migrants moving between Canada and the Caribbean. I focus on queer orientations and viral statuses as key nodes of subjectivity and/or sociality that may combine in different ways to produce (re)orientations (qua Ahmed 2006) and new social relationships—queer viral socialities—that generate migratory desires and journeys across transnational borders. However, queer HIV+ migrants from Global South locations like the Caribbean often encounter difficulties crossing Global North borders designed to facilitate entry for select categories of acceptable migrants. Acknowledging the productive yet unpredictable interactions of queer viral orientations and socialities that generate migratory desires and journeys alongside the harsh surveillance and disciplinary actions of nation-states’ border security regimes draws attention to the intersectionality and complexity of subjectivities, socialities, desires, and movements of queer HIV+ Caribbean migrants specifically, and transnational migrants more generally as they navigate the barriers and inequities of state migration apparatuses.

Deon was explaining to me why he decided to leave Jamaica and claim asylum in Canada: “It is bad being gay, but HIV and gay, that’s double jeopardy.” As a gay man, he had found everyday life in Jamaica challenging but tolerable. However, after learning he was HIV positive (HIV+), he became increasingly anxious about others discovering his status which would be, as he put it, a “catastrophe”. Fortunately, Deon had a partner in Canada who was able to obtain a visitor visa for him, and he knew other gay, HIV+ Jamaicans who had traveled to Canada and applied for refugee status, so “because of the persons I knew”, he arrived well prepared for the refugee claim process, bringing his professional and educational certificates and “documentations of those who had been murdered or killed in Jamaica…”. Deon believed that his claim for refugee status was successful because he was transparent about his HIV status, and he was well prepared to prove his gay identity and his experiences of persecution based on his viral status and identity. He now volunteered with a Toronto LGBTQ refugee support group helping others (in Jamaica and Canada) prepare their refugee claims. Looking back on his journey, Deon said, “HIV made me a different person...I look at life differently and I view people differently. If I wasn’t HIV+…I wouldn’t have met some people. In fact, I wouldn’t be in Canada.”

Throughout my research (Murray, 2016) examining the experiences of queer migrants who claimed asylum in Canada based on persecution in their country of origin due to their sexual orientation or gender identity expression (SOGIE)), a number of Caribbean interviewees identified their sexual orientation and HIV+ status in similar ways to Deon. For these people, sexual orientation and viral status were important factors contributing to the desire to migrate. In this paper I want to focus on queer orientations and viral statuses as key nodes of subjectivity and/or sociality that may combine in different ways to generate (re)orientations (qua Ahmed, 2006)^1^ of self-understanding and contribute to new social connections with other similarly identified people located within and beyond national borders, which may generate migratory desires. In other words, queer and viral (re)orientations contribute to the formation of queer viral socialities (Lorway, 2021; Murray, 2022) and migratory desires. Queer viral socialities contribute to the formation of social networks through which information about migration to queer and HIV supportive destinations is transmitted and transnational movements unfold.

However, I also introduce the narrative of Edward, a queer HIV+ Caribbean migrant, who challenges the predictability of the relationship between queer viral orientations, socialities and migrant desires and movements. His narrative illustrates how an HIV+ status and queer orientation does not necessarily generate re-orientations towards similar social networks nor does it always generate similar migratory pathways from the Caribbean to Canada, due to difficult encounters with the Canadian state’s border security and immigration apparatus. Thinking about queer and HIV as inter-related but unpredictable nodes of orientation and sociality in relation to migrant lives and immigration apparatuses contributes to better understanding how and why patterns of migration take place and expands our thinking of HIV beyond the dominant framework as a biomedical risk for “vulnerable” migrant populations.

In the rest of this article, I will first identify how HIV is configured in the field of queer migration research and how migration is configured in social science-based HIV/AIDS research in order to illustrate predominating conceptual frameworks that, for the most part, elide the social and subjective qualities of HIV, resulting in limited interpretations of HIV’s significance and meaning in relation to queer migrant populations. I then examine the intersection and impact of queer viral orientations, socialities, and migrant desires through excerpts from conversations with three Caribbean queer, HIV+ migrants, Latoya, Devon, and Edward. While some of these conversations reveal intersections of queer viral orientations, socialities and migratory journeys similar to Deon, others reveal important differences, particularly in relation to the discovery and disclosure of one’s HIV status and experiences with Canada’s immigration and border security apparatus, demonstrating that queer and viral orientations do not always align in the same way at the same moment for queer migrants, nor do they automatically generate identical queer viral socialities, migrant desires and movements. These diverse experiences complicate the significance of and relationships between queer viral orientations and socialities as these queer Caribbean migrants encounter the nervous immigration apparatus of the Canadian nation-state and its entrenched suspicion of racialized migrants from the global South who cross Canadian borders in unorthodox ways.

It should be noted that research participants discussed in this article self-identify and/or are identified by others (such as the Canadian Immigration and Refugee Board (IRB)) as Afro-Caribbean, Black, queer, queens, gay, or transgender. These are ethno-racial, sexuality and gender categories utilized in interviewees’ refugee claim applications or in conversations with me and may not necessarily represent their own categorizations or identifications.^2^ Also, this group of research participants’ border crossings fall under what the state deems “asylum seekers” or “refugee claimants,” and therefore represent just one sub-set of possible migration routes and categories. As will be explained below, queer migration is constituted through numerous intersectional orientations and statuses of queer migrants, multiple pathways of migration, and diverse encounters with border security regimes and immigration apparatuses of nation states.

## HIV/AIDS and queer migration research

Queer migration studies is a now well-established field of research that explores relationships between desire, identity, (im)mobility, and regimes of power and knowledge. This body of scholarship has been enabled by understanding sexuality as both an object of knowledge and subjective orientation constructed through intersecting relations of power (including race, ethnicity, gender, class, and citizenship status) operating on multiple scales (local, national, transnational). Queer migration studies reveals the fundamental ways in which sexuality simultaneously undergirds *and* undermines the organization and boundaries of nation states, and connects migration and sexuality to projects of nationalism, transnational capitalism, and neo-imperialism (Agathangelou, 2004; Bhagat, 2020; Carillo, 2004; Epps et al., 2005; Espin, 1999; Hart, 2002; Kuntsman, 2008; Luibhéid, 2002, 2008; Luibhéid and Cantú, 2005; Luibhéid and Chávez, 2020; Lewis and Naples, 2014; Manalansan, 2006; Mole, 2021; Patton and Sanchez-Eppler, 2000; Puar, 2007; Weston, 2008; White, 2013, 2014; Yue, 2008). This body of research also foregrounds the centrality of ongoing transnational ties between queer migrants and multiple homelands, and their impact on the social worlds of both old and new homelands (Carillo 2017; Cantú 2009; Decena 2011; Gopinath 2005; Manalansan 2003).

In the Canadian context, there is a well-established, diverse body of research examining issues of im/migration as it intersects with racism, gender, class, multiculturalism, nationalism, and citizenship (i.e., Dua, 2007; Iacovetta, 2000, Macklin, 2003; Thobani, 2007, Valverde, 2008). Thanks in part to the burgeoning field of queer migration studies, increasing attention has focused on the significance of sexuality as queer migrants encounter Canadian immigration policies, border security regimes, refugee bureaucracies, and discourses of citizenship (Jabr, 2021, Jenicek et al., 2009; Jordan, 2010; Karimi, 2020; Lee, 2018; Kinsman and Gentile, 2010; Lewis and Naples, 2014; Murray, 2015, Ou Jin Lee, 2018, Ou Jin Lee and Brotman, 2011; Ricard, 2014; White, 2013 & 2014, Wright, 2018). In the Caribbean context, transnational migration (forced and voluntary) is foundational to the constitution of Caribbean societies and continues to be a central component of contemporary life throughout the region. Research on the queer dimensions of Caribbean migration has expanded substantially over the past 20 years, focusing on queer Caribbean migrants’ socio-sexual cultures and identities, the impact of transnational sexual rights movements on queer migration, and complicated relationships with families and multiple “homes” (Attai, 2017; Calixte, 2005; Crichlow, 2004; Decena, 2011; Glave, 2008; LaFountain-Stokes, 2009; Kempadoo, 2004; McNeal, 2019; Mohabeer, 2019; Wahab and Plaza, 2009; Walcott, 2009).

However, for the most part, queer migration research in both regions has not investigated how HIV/AIDS intersects with sexual orientation, gender identity, and/or migrant desires, nor does it consider how HIV/AIDS impacts local and transnational queer migrant social worlds. Adam and Rangel’s research, (2015) is one of the few studies of transnational queer migrants (Latino gay men in Canada) to identify HIV status as a factor contributing to the decision to migrate, noting that some of their queer HIV+ respondents seek to migrate to what they believe is a destination with greater economic opportunities, a benevolent state system with a stable, well-funded public health infrastructure, and a social environment that is less discriminatory towards queer, HIV+ people (687-688). In their research examining young Senegalese gay men who flee to Mauritania seeking asylum through the United Nations High Commissioner for Refugees (UNHCR), Broqua et al. (2020) note that some diseases such as HIV seemed to be an asset in the asylum process as a higher proportion of HIV+ gay Senegalese men were resettled by the UNHCR compared to HIV negative gay men, indicating the possibility of HIV status as a motivational factor in migratory desires. However, they also report a high number of deaths from AIDS among this group, highlighting the increased risks of migration for people living with HIV (PLHIV).

In the vast field of social science research on HIV/AIDS, migration has been identified as a key factor in understanding epidemiological patterns of infection since the earliest days of the pandemic over 4 decades ago. In their edited volume examining mobility, sexuality, and AIDS, Thomas et al. note the longstanding concern about mobility and infectious diseases that was, at the beginning of the pandemic, primarily framed in terms of fear of (im)migrant populations introducing and spreading diseases to local populations (2009:2). These “fear-based” representations of the sexual cultures of migrant populations have been substantially critiqued, and much of the literature on HIV, sexuality and migration over the past 20 years has shifted to focus on (1) sexual cultures and/or identities and HIV-related risks and vulnerabilities of mobile populations, the communities that host them, and the communities to which they return or (2) HIV-related stigma and discrimination experienced by migrant populations in destination/host locations (i.e., Infante et al., 2009; Manalansan, 2006; Thomas et al., 2009). In much of this research population mobility is primarily framed as a “key driver” of the HIV pandemic with a focus on how and why migrant populations are “vulnerable” due to experiences of intersectional discrimination or “at risk” due to sexual behaviors that increase the possibility of HIV transmission (these two factors are often linked; see Deane et al., 2010; Infante et al., 2009). Thus, in most HIV/AIDS research, the sexual cultures of migrant populations continue to be framed primarily in terms of “risk” and “vulnerability” to HIV infection or stigma, with little attention given to the social dimensions or affects of HIV.

The social affects of HIV have been well documented in research emphasizing biosociality, “the notion that people with shared biological conditions come together to form social networks” (Bradley, 2021:544). In HIV/AIDS research, a particular formation of biosociality has been identified as “viral socialities … the affective, intimate ties that form between groups of people as they confront the lived effects of denigration, deprivation, and injustices” attributable to an HIV positive (HIV+) status (Lorway, 2021: 217; see also Mazanderani, 2012; Murray, 2022). In this paper, I emphasize how the viral sociality of HIV, or the social dimensions of HIV, are produced through (re)-orientations generated through the combination of queer subjectivity and an HIV+ status, which may then generate migrant desires. In other words, an HIV+ status may contribute towards a subjective shift or reorientation of desires, feelings and thoughts about oneself in relation to past, present, and future temporalities, which may generate migratory desires. Feelings and desires related to health and well-being (material, physical and emotional), social relations (partners, families, and communities), and/or educational and employment opportunities, may be reoriented through diagnosis of an HIV+ status, which may generate desires to migrate to places where one imagines their reoriented subjectivity will be achieved, accepted or supported. However, as we shall see below, while an HIV+ status *may* contribute to the establishment of new orientations, social relations, and migrant desires, this is not a given.

## Queer viral socialities and migrant desires: 3 journeys

The following narratives are derived from formal interviews and informal conversations with three Barbadian queer migrants, two of whom I have known since 2004, and one of whom I met during fieldwork in Barbados in 2019. I met these individuals in either Toronto, Bridgetown Barbados, or both locations over the course of three distinct, but overlapping research projects carried out over a 15 year period, the first examining homophobia and queer life in Barbados (Murray, 2012), the second examining SOGIE refugees as they moved through the Canadian refugee determination process (Murray, 2016) and the third investigating how people living with HIV in Canada and Barbados are navigating hegemonic “end of the HIV crisis” proclamations and decreasing public interest in and funding of HIV prevention and support programs (Murray, 2021).

While it might seem rather obvious, it is nonetheless important to prelude these narratives by pointing out that none of these queer migrants began their lives thinking of themselves as migrants or refugees, and in fact for quite a few, “refugee” was a relatively new identity term imposed upon them by the process through which they migrated across national borders and into the Canadian government’s immigration apparatus. This obvious point—that most queer migrant refugees only recently came to recognize or acknowledge this government imposed status—challenges any singular representation of the category “queer migrant or refugee” and reminds us that queer viral orientations and socialities do not align with any one specific migrant category.

*Latoya and Devon:* I first met Latoya and Devon at a support group for LGBT youth in 2004 when I was conducting fieldwork on homophobia and queer life in Barbados (Murray, 2012). Latoya was in her upper-teens and identified by her friends as a “queen,” a fluid term with a variety of meanings, but generally referring to someone identified as “male” at birth who desires cisgender men, and performs, dresses, or speaks in ways that are socially interpreted as feminine. At that time, Latoya told me that she wanted to spend more time “as a woman”, although she was still dressing in men’s clothing and presenting as a man when she went to school or walked the streets during the day.^3^ Devon was older, in his early thirties, and self-identified as “gay” and a “queen”. He was also one of the few members of the support group who was open about his HIV positive status. Devon and Latoya had been friends for several years.

Most of Devon’s and Latoya’s social life was spent with other queens, trans-women, and gay men who attended the support group meetings, visited local bars and parties together, and generally hung out with each other. Devon and Latoya came from poor or working-class neighborhoods in and around Bridgetown and gravitated to the support group because its founder and president was one of the few public voices of “Barbadian gays and lesbians” at that time, and had a reputation for helping queer youth who were having problems with their families. In 2004, Latoya was still living with her mother and father, who accepted her as she was. Latoya wanted to leave school and look for work, but she said jobs were difficult to find. One of her friends told me that Latoya occasionally “worked the streets” (engaged in sex work), although Latoya did not acknowledge this to me. Despite the challenges she faced, I thought that Latoya was relatively content in Barbados. She had friends, a support group, and supportive parents. Devon, on the other hand, had faced a great deal of strife from his family when they learned of his sexual orientation, and he had left his family home at a relatively young age. He had moved around Bridgetown, often renting a room in a house or apartment with other queens, transgender women, and/or gay men. In his early 20s (in the late 1990s) Devon became very ill from AIDS—he told me that he had not known his status until he had a major seizure and ended up in hospital. Devon’s illness resulted in some long-term physical disabilities which made work challenging, and he was living on a disability pension in a house with some other queens.

Devon was one of a few support group members who asked me about “gay life” in Canada at that time (2004), and indicated he was thinking about migrating because he had heard life was easier there. However, others like Latoya did not seem interested in leaving and told me that they had heard life there could be difficult for other reasons (racism, homophobia, weather, and jobs). Seven years later, in 2011, I was surprised to see Latoya and Devon walk into a Toronto SOGIE refugee support group meeting, although I think they were just as surprised to see me. Devon looked relatively unchanged since I last saw him, but Latoya was more performatively feminine—she was wearing a tight black mini-skirt, a purple faux angora sweater, high heels, with highly skilled make-up applied to her face, and her eyes shone with bright blue contact lenses.

When we met after the meeting, Latoya told me how, over the past few years, she had entered some (gay/queen) beauty pageants in Barbados and Trinidad and was really enjoying the whole experience, but everyday life in Barbados had been getting worse, particularly as she was now trying to live as a woman “full-time”. She also had a relationship with a man who had eventually become violent, so she broke up with him, but she continued to live in fear of being attacked by him. For Devon, life in Barbados continued to be challenging, as he found his disability pension difficult to live on, and he and his queer roommates had to keep finding new accommodations due to harassment from neighbors or landlords asking them to leave.

Devon and Latoya said that in their social networks, they knew some queens who had traveled to Canada, claimed asylum and “made it through”. They contacted Gigi, one of the first queens to move to Toronto, who told them that life was easier in Canada where there was free healthcare, social assistance, and people were more accepting of people like “us”. Gigi provided advice on how to claim asylum and offered to help them when they arrived in Toronto. Latoya’s mother and auntie paid for her ticket to Toronto, and after arriving, she went straight to Gigi’s apartment (Barbadians do not require visas to visit Canada, which generally results in easier movement through Canadian customs). Devon first flew to New York where he had a friend, and then traveled to Toronto via bus. He also went immediately to Gigi’s apartment (Devon arrived in Canada a few months before Latoya). A couple of days after arriving, Gigi gave them the address of a Canada Immigration and Citizenship office in a Toronto suburb and told them what to say when they arrived there. Gigi also put them in touch with the lawyer she had worked with in her successful asylum claim and told them to start attending the SOGIE refugee support group meetings at the LGBTQ community center in downtown Toronto (where I met them).

When I asked if other queer Bajans were making similar journeys, Latoya said, “When I was staying with Gigi, Devon was staying (there) too, and then other people (were) coming...first it was Tamar and her boyfriend, then 3 other people coming to do the same thing…” Devon agreed, saying, “There was 2 first, then Jada, then 2 young boys, that’s 4...then Savannah, her boyfriend, David…Vanessa, Adrian…about 10 (so far)”. With these ongoing arrivals, Gigi’s apartment became too crowded for Latoya and Devon, so they moved out (separately) and each ended up couch-surfing through several queer Bajans’ homes in and around Toronto. However, a few months later Latoya suddenly collapsed, which put her in the hospital where she was told she had AIDS.^4^ After she recovered, she moved to Covenant House, a shelter for homeless youth, where she was assigned a social worker who helped her find permanent accommodation as she waited for her refugee claim hearing. Devon also eventually found more stable long-term accommodation in a town outside of Toronto.

A few months later, in January 2012, I attended Latoya’s refugee claim hearing at the IRB offices in downtown Toronto. She was there with her lawyer, Antonio, and a social worker. While we sat in the waiting area, Latoya informed me that Devon had his hearing a few weeks ago and received a positive decision, which made her feel hopeful, although she was still very nervous. During the hearing, the IRB Member (the individual responsible for making a final decision on Latoya’s application) asked her several questions, including “who and what” she feared if she returned to Barbados. Latoya responded, “I fear the public and the police force…I’m afraid being transgender in Barbados, the police make sport of it.” Latoya’s lawyer Antonio then asked her the following questions:

A: When did you find out about the refugee program?

L: 2010; in February, Gigi…I told her about my attack, and she said that I should come and when I was coming to call her…

A: What’s your health status

L: I’m HIV+

A: You found this out where?

L: In Canada…after I got here

A: What are attitudes to HIV positive people there (in Barbados)?

L: You’re scarred, people don’t want nothing to do with you…If you’re gay and transgender, (and) you’re HIV+; people come up and say you’re wrong, you need to go to church…

A: If you’re forced to return to Barbados what will happened

L: I’ll be killed

A: Why

L: I’m transgender and HIV+ …my death won’t be researched by police

Following these questions, the Member ended the hearing and after a brief recess, rendered a positive decision. LaToya started to cry heavily, saying this had all been too much. Later, she said she was overwhelmed by the combination of stress and relief from the hearing and the decision.

When I saw Latoya and Devon a few months later on Church Street in Toronto’s gay village, they said that they were doing ok but they were still looking for work, and they wanted to move out of their respective accommodations to their own apartments, but due to high rental costs in Toronto, that was not an option. I lost contact with them soon after (phone plans and numbers changed frequently), but one of their friends who I stayed in contact with said that Devon continues to live outside Toronto, primarily relying on the Ontario Disability Support Program, and LaToya lives in a Toronto apartment working as a seamstress and make-up artist. However, the friend said that LaToya recently had some health issues resulting in hospitalization and that she was experiencing mental stress and was considering returning to Barbados.

*Edward:* I met Edward in 2019, during fieldwork for research examining how Barbadian PLHIV were navigating decreasing public interest in and funding of HIV/AIDS services (Murray, 2022). Edward identified as “gay” and was in his late 40s. He left Barbados in the mid-1990s for Toronto where he had a friend he could stay with. He did not apply for a work or residence visa, so most of his jobs were “under the table” such as waitering or kitchen work. However, he was caught by Canadian Border Security Agency officials, jailed and deported. A couple of years later, he used fake papers to return to Toronto, where he once again found some work, but then became sick and found out he was HIV+. He was able to use his friend’s “social” (I assume this meant social insurance number or health insurance card) to get on a free HIV medication program. Around the same time (2000–2001), he heard that some gay men from Jamaica had successfully applied for refugee status based on persecution due to their sexual orientation and HIV status, so he submitted a claim, which indicated that he was gay and HIV+. However, his hearing, held over a year later, resulted in a negative decision: “they said Barbados was not… … violent enough for (people like me)…to apply for that.”. When I said how unfair this must have felt, given that other queer, HIV+ Bajans who applied for refugee status were being accepted, he laughed ruefully: “I’m telling you! Like… nearly everybody is getting tru now, and I was like… I just said to myself, ahhhh, I just born, I’m born with bad luck so that’s just how it is. I didn’t get tru.”

Edward’s friends urged him to petition the IRB’s negative decision, but by this time, Edward had become involved in selling and using drugs and realized he was not on a good path:

“I…was coming down to de last stages, I got involved in like, drugs and shit, yeah. So it was like… I was on crack/cocaine. So that’s another reason too that made me… I decided I got to get away from Toronto anyways, because I was like going down…So I came back. I say I gotta get away from that….I ain’ about to try to fight it or nothing like that, I just wanted to get away from it…”

Edward was scared about returning to Barbados due to being gay and HIV+ and the discrimination that could result if people found out. At that time (the early 2000s) Edward said “the stigma is double (with) the gay thing plus the HIV…“. When he returned, an ex-boyfriend helped connect him to a clinic and doctor specializing in HIV, however, things became complicated as he had stopped taking his HIV meds while using drugs in Toronto, so those meds no longer worked and they had to find another HIV pharmaceutical regimen. This was a difficult period for Edward, and he started to use drugs again, he was then caught by police, and he ended up being incarcerated for “some months” at Dodds Prison, located outside of Bridgetown. Nevertheless, throughout all of this, his family (mother and sister) were supportive even though they learned of his HIV status through one of his (now former) friends.

Over time, Edward’s health stabilized, and now things were “alright,” but he was frustrated over the lack of employment opportunities, the welfare support in Barbados was “bullshit,” and there was very little support for PLHIV. Edward continued to think about and look for jobs: “I want two popcorn machines, but I can’t (get them) as the collateral is not coming because… (of) my incarceration…and de police record.” Whenever he applied for a job they would “dig up” his police record and he would have to explain over and over, “I done all the charges there….How can I get a proper job…I gotta settle for a job that don’t ask for police character… So I need to have my own thing but I don’t…”.

Edward had heard about some PLHIV support groups in Barbados, but didn’t know anybody in them, so he didn’t bother to find out more. To him they seemed, “kinda like hush hush like… or… like these people (in them) is like kinda friends of friends…I’s prefer to stay with myself and do my own thing. Try to survive my own way.” He knew a few other LGBTQ people in Barbados, although “a lot of my people that I’d known before (I left) died”. He felt that Barbadian society today was less discriminatory towards gay and HIV+ people than before, but “people still make a lot of jokes”. His mantra for dealing with that attitude was “I’m too blessed to be stressed.”

## Queer viral socialities, colonial legacies, and nervous nation states

These condensed narratives of a small group of queer, HIV+ Caribbean migrants convey the complex commingling of queer and viral orientations and socialities with migrant desires and journeys. For Deon, Latoya, Devon, and Edward, queer orientations and queer social networks were foundational to everyday life in their Caribbean societies. These queer social networks were intimate and social, local, and transnational–they were the source of friendships and sexual and romantic relationships, but they were also conduits through which information about shelter, work, health, and/or overseas travel and opportunities could be obtained. They were maintained through shared everyday spaces of homes, streets, and bars, and through digital technologies and social media like cell phones, computers, WhatsApp, and Facebook. For the purposes of this article, I have focused on the transnational dimensions of these queer social networks that generate and sustain queer migrant desires: Latoya and Devon knew Gigi in Toronto; Edward and Deon had queer Caribbean friends in Toronto. Transnational connections with friends and lovers overseas conveyed information about queer life elsewhere and how to cross international borders with limited means, which led some (but not all) of this group to desire migration based on the desire to be with loved ones, better job prospects, the promise of improved health support systems, and/or the appeal of larger, more visible queer communities. Migratory desires were thus generated and sustained through queer orientations and transnational queer social networks.

For everyone in this group, an HIV+ diagnosis shifted subjective orientations at the very least in terms of understanding one’s health status and one’s relationship to health care systems, but also in other, more unpredictable ways. People discovered their HIV status at different points in their migratory journeys, rendering its relationship to migratory desires less predictable. For people like Devon and Deon who were diagnosed with HIV while still living in Barbados and Jamaica, an HIV+ status contributed to the desire to migrate, as they experienced or feared the “double jeopardy” of queer and HIV discrimination in their respective societies, and they heard from other queer HIV+ Caribbean migrants that better health care, support, and acceptance could be found in Canada. For them, queer orientations and socialities intertwined with or were augmented by viral orientations and socialities which, together, contributed towards migratory desires and journeys.

However, for other queer migrants like Latoya and Edward, who were diagnosed after arriving in Canada, an HIV+ status generated different orientations and effects: Latoya’s discovery of her HIV status after hospitalization in Toronto didn’t impact her social networks or migratory desires but operated as a central component of her refugee claim (similar to Deon), and possibly contributed towards her successful refugee claim and trajectory towards obtaining Canadian citizenship. Edward’s HIV+ status (also diagnosed in Toronto) did not help his refugee claim, as the IRB Member told him that Barbados was not violent (discriminatory) enough for “people like him” to apply for refugee status. Edward’s application for refugee status took place in the early 2000s (about 10 years prior to Latoya’s claim), at which time the SOGIE refugee category was relatively new to the IRB so adjudicators may have been less familiar with gender and sexual discrimination in the Caribbean region. On the other hand, Rehaag (2019) has shown substantial variation between IRB adjudicators’ acceptance and rejection rates, so Edward’s claim may have been assessed by an adjudicator with a high rejection rate, revealing the arbitrary (and inequitable) nature of the refugee determination process in Canada, and lending weight to Edward’s claim that he had bad luck.

Edward’s unsuccessful claim (which could have been appealed), combined with his increasing drug use and an unstable connection to health care (due to his ‘illegal’ status in Canada), led to his decision to return to Barbados, but life back home proved to be difficult, and he started to use drugs again and ended up serving time in prison. 20 years after his HIV diagnosis, life was more settled for Edward but it still wasn’t easy. Despite long-term connections with queer social networks in Canada and Barbados, Edward chose to keep his HIV status relatively private in Barbados, where only his immediate family knew. Thus, while Edward’s queer orientation and social connections contributed to migratory desires and journeys, his viral status, combined with difficult experiences of the Canadian immigration apparatus and drug use, generated different movements (migrating back to Barbados) and different socialities as a returned, then incarcerated, Barbadian citizen.

Therefore, while the diagnosis of an HIV+ status had a significant impact on all participants, generating a reorientation towards one’s past, present and future decisions, relationships, and desires, it did not automatically generate similar socialities and sometimes heightened anti-social desires (non-disclosure) based on fears of discriminatory treatment and ostracism. Furthermore, Edward’s narrative demonstrates that an HIV+ status does not always generate unidirectional migratory desires away from Global South locations like Barbados towards Global North destinations like Canada and may in fact generate opposite movements. His encounters with the Canadian state’s border security and immigration apparatus, (CBSA officers, detention centers, immigration offices, and/or IRB adjudicators), generated frustration and stress, and contributed to his decision to return to Barbados. An HIV+ status therefore occupies a less predictable, more ambiguous position in relation to queer viral orientations and socialities and migrant desires and journeys.

Despite the specificity of desires and movements generated through queer viral orientations and socialities, these narratives also reflect features common to most Caribbean “transmigrants,” “immigrants whose daily lives depend on multiple and constant interconnections across international borders and whose public identities are configured in relationship to more than one nation-state” (Schiller et al., 1995).^5^ Queer HIV+ Caribbean migrant journeys are not determined solely through transnational queer Caribbean social networks—they are also the result of generations of transnational migration between the Caribbean region and nation-states connected through colonial histories and networks, making destinations like Canada more accessible and/or administratively less difficult to enter. These journeys are also aided and abetted through other socialities such as family or kinship networks (i.e., Latoya’s mother and auntie purchasing her airfare to Toronto), underscoring Trotz’s observation of Caribbean transnational migration constituted through “ongoing relations between home and away, whether articulated in the material and emotional support of family members…” (2006:43).^6^

Finally, it is important to underscore how these queer HIV+ Caribbean migrant encounters with border security and immigration apparatuses align with queer migration scholarship’s arguments about the legacies of colonialism, transnational capitalism, slavery, forced transportation, and exile shaping present-day queer migrations, and how queer migrants comprise “impossible subjects” with unrepresentable histories that exceed existing legal categories of the nation-state.^7^ Complex intersections of queer viral orientations with other social identifications (race, gender, and class) generate complicated crossings of national borders revealing the latter’s limited categories of admissibility and perpetual nervousness about migrants whose complex identities exceed or transgress “acceptable” migrant classifications and categories. (Luibheid, 2008:171, 176). The incarceration and deportation of queer, racialized, and poor migrants like Edward reflect the heteronormative, racist and classed legacies of colonial orders and ongoing structural barriers that exclude and/or discipline marginalized people on the move.

## Conclusion

While it is axiomatic to state that a queer orientation produces a different relationship to social worlds predicated on heteronormativity (Ahmed, 2006) and that an HIV+ status invokes a reorientation towards social worlds predicated on fear of infection and illness (Lawson et al., 2006), in this paper I have tried to illustrate the importance of investigating how these orientations and statuses interact and contribute towards the production of migrant desires in unpredictable ways, providing new insights into research on HIV/AIDS and queer migration. Queer migration research has clearly established the centrality of queer orientations and socialities in relation to migratory desires, but viral status, and its interaction with queer orientations, socialities and migrant desires has yet to be thoroughly investigated. HIV/AIDS research has clearly identified migrant populations as vectors of HIV risk or vulnerability but has restricted the significance of HIV/AIDS to the realm of the biomedical and has not extended some of the important insights of HIV biosociality to transnational migration. Acknowledging the productive, yet unpredictable, interactions of queer HIV orientations and socialities in relation to migratory desires and journeys alongside the harsh surveillance and disciplinary actions of nation-states’ border security regimes draws our attention to the complexity and unpredictability of intersecting subjectivities, socialities, and desires of transnational migrants as they encounter the structural barriers and inequities of state border security and immigration apparatuses.

The queer orientations of the participants featured in this article generated attachments to and connections with longstanding local and diasporic queer Caribbean social networks, which sometimes, but not always, generated migratory desires to destinations like Canada. However, these migratory desires are not solely structured through queerness, as they are also connected to long-established routes of Caribbean in and out migration forged through transnational kinship and community connections which are structured through the legacies of colonial diasporas such as the British Commonwealth.

For these queer Caribbean migrants, the diagnosis of an HIV+ status generated re-orientations related to health, relationships, and present and future plans, but it was less predictable in relation to generating social networks and migrant desires. If diagnosed in the Caribbean prior to migration, an HIV+ status may generate or intensify migratory desires and create or intensify connections with other queer HIV+ migrants sharing in order to obtain support and information pertaining to migration, refugee claims, and/or health care systems. If diagnosed in Canada, an HIV+ status may generate similar social connections or it may be utilized as a social identity marker through which refugee status is claimed. However, the disclosure of one’s HIV status via a refugee claim does not automatically result in a successful claim, nor do all queer migrants assume disclosure will automatically generate positive social connections, so some individuals choose not to disclose their status and/or connect to PLHIV networks.^8^ In fact, for some queer migrants like Edward, an HIV+ status, in combination with ongoing difficulties with the Canadian immigration apparatus, leads to the desire to return to the Caribbean. Thus, an HIV+ status generates less predictable socialities, desires, and journeys.

Throughout these narratives, the security and immigration apparatus of the Canadian nation-state challenges and disrupts migrant plans, journeys and settlement. While some queer HIV+ migrants like Deon, Devon, and Latoya utilized queer viral orientations and socialities to their advantage, obtaining information on how to successfully navigate the complexities of a complex and often hostile bureaucratic apparatus, others like Edward did not, resulting in incarceration and/or deportation. Edward’s migratory journeys were formed through queer orientations and socialities, but due to his limited resources and/or knowledge about the rules and regulations of the Canadian immigration apparatus, he crossed borders in a way that attracted the security and surveillance components of the apparatus and then later submitted a refugee claim that was deemed “not credible” by an IRB adjudicator, rendering settlement so difficult that, in the end, returning to Barbados was easier than ongoing struggles with a hostile Canadian state.

Edward’s experiences of incarceration and deportation are not unique (see, e.g., Bruemmer, 2021; Contreras, 2019; Mann, 2018; Paperny, 2021; Shakhsari, 2014) and remind us that while Caribbean queer viral orientations and socialities may generate migrant desires, the journeys generated from these desires are unpredictable in their movements and outcomes, particularly for those with limited financial means and/or knowledge of a complex immigration apparatus that operates with rigid categories and definitions of admissibility and/or credibility. The unorthodox journeys and complex life experiences of racialized queer HIV+ migrants from the global South like Edward generate heightened surveillance and punitive actions by the nervous nation-state which supports the migratory dreams and desires of a few, but ruins many others.^9^

## Notes

1. When discussing sexual orientation, Ahmed states, “I want to offer a phenomenological approach to the very question of what it means to ‘‘orientate’’ oneself sexually toward some others and not other others.” (2006:68). In this paper I address how sexuality and viral status may interact to produce subjective and social (re)orientations and (re)alignments, resulting in unpredictable migratory desires.

2. For more on distinctions and troubled relationships between hegemonic western and non-western sexual orientation and gender identity terminologies, see Murray, 2016

3. Latoya did not identify as “transgender” in conversations with me at this time, although she may have self-identified with that term.

4. From our discussions it appeared that Latoya learned of her HIV status when she was in hospital, however, all refugee claimants must undergo a mandatory medical exam as part of the refugee application process (Bisaillon and Ells, 2014). The exam includes an HIV screening test, so it is possible that Latoya could have been informed of these results and known her status prior to hospitalization.

5. See Trotz (2006) for a critique of the tendency in transmigrant research to emphasize movements between “home” and “host” countries, ignoring the possibility of multiple movements and locations of Caribbean transmigrants.

6. Not all queer Caribbean transmigrants maintain strong transnational family/kinship ties: For some the desire to migrate is generated in part through experiences of discrimination, violence and/or ostracization from families and communities.

7. Queer migration scholarship also recognizes how queer transnational social networks are maintained through social media and digital technologies in which friendships, relationships, advice, and strategic information are exchanged (see, e.g., Bayramoğlu and Lünenborg, 2018; Patterson and Leurs, 2019).

8. See De la Cruz et al., 2020 for similar findings on HIV status disclosure among sub-Saharan African immigrants living with HIV in western Canada.

9. The nation-state’s ability to “give life” to some queer migrants while “taking life” from others (literally and figuratively) through its border security and immigration apparatus contributes towards what some scholars refer to as “queer necropolitics.” For more on this concept see Bhagat, 2020, Broqua et al., 2020, and Murray, 2016).
